# *Artemisia Capillaris* leaves inhibit cell proliferation and induce apoptosis in hepatocellular carcinoma

**DOI:** 10.1186/s12906-018-2217-6

**Published:** 2018-05-08

**Authors:** Juyoung Kim, Kyung Hee Jung, Hong Hua Yan, Min Ji Cheon, Sunmi Kang, Xing Jin, Sunghyouk Park, Myung Sook Oh, Soon-Sun Hong

**Affiliations:** 1 Department of Biomedical Sciences, College of Medicine, Inha University, 3-ga, Sinheung-dong, Jung-gu, Incheon, 400-712 Korea; 2 Natural Product Research Institute, College of Pharmacy, Seoul National University, Sillim-dong, Gwanak-gu, Seoul, 151-742 Korea; 3 Department of Oriental Pharmaceutical Science, College of Pharmacy, Kyung Hee University, 26, Kyungheedae-ro, Dongdaemun-gu, Seoul, 02447 Korea; 4 Department of Biomedical Sciences, College of Medicine, Inha University, 366, Seohae-daero, Jung-gu, Incheon, 22332 Republic of Korea

**Keywords:** *Artemisia capillaris*, Apoptosis, Xenograft, HCC, PI3K

## Abstract

**Background:**

Natural product is one of the most important sources of drugs used in pharmaceutical therapeutics. *Artemisia capillaris* has been traditionally used as a hepatoprotective and anti-inflammatory agent. In this study, we extracted an ethanol fraction (LAC117) from the dried leaves of *Artemisia capillaris* and identified its anticancer activity and mechanism of action against hepatocellular carcinoma (HCC).

**Methods:**

Anti-proliferative effect of LAC117 was evaluated by MTT assay and BrdU assay. The apoptotic effect of LAC117 on the expression of cleaved PARP and cleaved caspase-3 was evaluated by Western blot and immunohistochemistry from in vivo mouse xenograft, respectively.

**Results:**

We found that LAC117 strongly suppressed the growth and proliferation of human HCC cell lines (HepG2 and Huh7). Induction of apoptosis was evidenced by the increases of cleaved caspase-3 and PARP as well as TUNEL-positive cells. Additionally, the pro-apoptotic effect of LAC117 was observed by a decrease in the expression of the XIAP and an increase in cytochrome *c* releases via mitochondrial membrane potential. Moreover, it significantly inhibited PI3K/AKT pathway in HCC in vivo and in vitro. LAC117 suppressed tumor growth in an ex vivo model as well as in vivo mouse xenograft by inducing apoptosis and inhibiting tumor cell proliferation.

**Conclusions:**

The present study highlights that LAC117 could not only efficiently induce apoptosis, but also inhibit the growth of human HCC cells by blocking the PI3K/AKT signaling pathway, suggesting that LAC117 would be a potentially useful drug candidate against HCC.

**Electronic supplementary material:**

The online version of this article (10.1186/s12906-018-2217-6) contains supplementary material, which is available to authorized users.

## Background

Hepatocellular carcinoma (HCC) is the sixth most commonly diagnosed cancer and the third leading cause of cancer-related deaths in the world [1]. Although conventional anticancer drugs such as sorafenib and doxorubicin have been used for the treatment of HCC, their toxicity and tolerance prevent long-term use [2]. Recently, natural anticancer drugs have been considered as alternative medicines because of their safety associated with long-term exposure [3]. Traditional herbal medicines have been widely used for HCC prevention and treatment because of their multi-targeted and coordinated intervention effects, and their significant anti-cancer activity has been identified [4, 5]. Natural plants, including *Pulsatilla koreana* and *Petasites japonicus* have already been shown to suppress the growth of HCC cells through modulation of cell proliferation, differentiation, apoptosis, angiogenesis as well as several signal transduction pathways [5, 6]. The efficacy of several natural products in cancer has been tested by clinical intervention trials that support the potential utility of these agents in the cancer prevention, treatment, and management regimens [7].

*Artemisia s*pecies have been used as food additives and traditional herbal medicines, particularly in various diseases such as cancer, inflammation, malaria, hepatitis, and microbial infections [8–10]. Among *Artemisia* species, *Artemisia capillaris* (AC) showed the anti-inflammatory effects in atopic dermatitis, chronic hepatitis B virus infection, and liver cirrhosis [11, 12]*.* Also aqueous extract of AC has been shown to inhibit interleukin-1 receptor (IL-1R)- and tumor necrosis factor receptor (TNF-α)-induced cytotoxicity and ethanol-induced apoptosis of liver cells [13]. In addition, AC inhibited inflammatory response through preventing NF-kappa B activation in HCC cells [14]. In addition to the anti-inflammatory effects of AC in cancer, its anticancer capacity has recently been reported in different type cancers. Indeed, AC inhibited cell growth and induced apoptosis in breast cancer and leukemia [15, 16]. Moreover, the major constituents of AC such as capillin and scoparone exhibit anti-cancer effects in breast, prostate, lung, and liver cancers [17–19]. However, there have been no previous studies evaluating the anti-cancer effect of AC leaves in vitro and in vivo models of HCC. In this study, we newly extracted an ethanol fraction (LAC117) from the dried leaves of AC and investigated its anticancer activity and mechanism of action against HCC.

## Methods

### Chemicals and antibodies

Primary antibodies against cleaved PARP (cat.n.9541), cleaved caspase-3 (cat.n.9661), XIAP (cat.n.2042), p-AKT (cat.n.4060), p-GSK3β (cat.n.5558), p-mTOR (cat.n.2971), and β-actin (cat.n.4970) were purchased from Cell Signaling Technology (Danvers, MA), PCNA (cat.n.ab29) from Abcam (Cambridge, MA), and cytochrome *c* (cat.n.13156) from Santa Cruz Biotechnology (Dallas, CA).

### Sample preparation of the LAC117 fraction

The dried leaves of *Artemisia capillaris* were purchased from Jung Do Herbal Drug Co. (Gyeonggi Province, Korea) and the voucher specimen (DBH16011101) was deposited in the Herb Resource Bank of Traditional Korean Medicine (http://herb-bank.com), Kyung Hee University (Seoul, Korea). The dried material (5 g) was extracted with 50 mL of 70% ethanol for 24 h at room temperature. Next, the extract was filtered, concentrated on a rotary vacuum evaporator, and completely freeze-dried (yield: 7.12%). The powder was stored at 4 °C.

### Chromatographic conditions of HPLC-MS analysis

An Agilent 1100 series HPLC system (Agilent Corp., Santa Clara, CA) was used to acquire chromatograms. All the chromatographic analysis was performed on a Phenomenex Kinetex C18 column (100 mm × 4.6 mm i.d. 2.6 μm). The mobile phase was composed of 0.1% formic acid in distilled water and 0.1% formic acid in methanol. The conditions of solvent gradient elution were 30% in 0–2 min, 30–90% in 2–12 min, 90% in 12–22 min, 90–30% in 22–22.1 min, 30% in 22.1–30 min, at a flow rate of 0.5 mL/min. The column temperature was maintained at 40 °C, and all the injection volumes of sample solutions were fixed at 2 μL. The eluent was directed to an ESI-LTQ-XL-Linear Ion Trap (Thermo Scientific) mass spectrometer and the data was acquired in full-scan and positive mode with mass range from 100 to 800 m/z.

### Cell culture

HCC cells (Huh7 and HepG2) were purchased from JCRB (Shinjuku, Japan) and American Type Culture Collection (Manassas, VA), respectively. Huh7 cells were cultured in Dulbecco’s Modified Eagle’s Medium (DMEM), and HepG2 cells were cultured in Minimum Essential Media Eagle (MEM) supplemented with 10% heat-inactivated fetal bovine serum (FBS, cat.n. 26,140–079) and 1% penicillin/streptomycin. FBS and all other reagents used for cell culture were purchased from Invitrogen (Carlsbad, CA). The cultures were maintained at 37 °C in an incubator with a controlled humidified atmosphere composed of 95% air and 5% CO_2_.

### Measurement of cell viability

Cell viability was determined using an MTT assay. In brief, cells were seeded at a density of 4 × 10^3^ cells/well in 96-well plates, followed by overnight incubation. On the following day, the media were removed, and the cells were treated with either vehicle as a negative control or various concentrations of LAC117 (1–100 μg/mL) and incubated for 72 h. After incubation of respective time, 10% of an MTT solution (2 mg/mL, Sigma, cat.n.M2128) was added to each well, and the cells were incubated for another 4 h at 37 °C. The formazan crystals were dissolved in DMSO (100 μL/well, Sigma, cat.n.D2160) with constant shaking for 5 min. The absorbance of the plate was then read with a microplate reader at 540 nm. Three replicate wells were evaluated for each analysis.

### Measurement of cell proliferation

To measure the cell proliferation activity of LAC117 in Huh7 and HepG2 cells, 8 × 10^3^ cells were plated per well onto 96-well plates. Following overnight culture, LAC117 was added at specified concentrations. After 24 h of incubation, cell proliferation was measured with a BrdU assay kit (Cell Signaling, cat.n.6813) per the manufacturer’s instructions. Plates were read at 450 nm by using a spectrometer.

### Western blotting

The cells were washed with DPBS before being lysed in a lysis buffer containing protease and phosphatase inhibitors. Equal amounts of proteins were separated using 8, 10 or 12% sodium dodecyl sulfate (SDS)–polyacrylamide gel electrophoresis and transferred onto polyvinylidene fluoride (PVDF) membranes. Protein transfer was confirmed using a Ponceau S staining solution (Sigma, cat.n.P7170). The blots were then immunostained with the appropriate primary antibodies (1:1000) followed by appropriate secondary antibodies (1:5000) conjugated to horseradish peroxidase. The primary antibodies specific to the interested proteins were used and detected manually using an X-Ray film by enhanced chemiluminescence (Amersham Biosciences, Piscataway, NJ).

### TUNEL staining

Huh-7 and HepG2 cells were plated onto chamber slides at a density of 5 × 10^4^ cells per chamber. At 24 h post-incubation, cells were treated with LAC117 (100 μg/mL) at 37 °C for 24 h. Coverslips with adherent cells were fixed in 4% paraformaldehyde (PFA) for 15 min at room temperature, and then rinsed in distilled PBS and incubated with equilibration for 1 min. TUNEL assay was subsequently performed by using a TUNEL kit ApopTag® Peroxidase In Situ Apoptosis Detection Kit (Merck Millipore, Temecula, CA. cat.n.S7100) in accordance with the manufacturer’s instructions. Huh7 and HepG2 cells were plated on 18-mm cover glasses for 24 h and treated with LAC117 (100 μg/mL). Apoptotic cells were visually identified in 10 randomly selected fields and photographed at a magnification of × 200. Apoptotic cells were counted to calculate the percentage of TUNEL-positive cells.

### Detection of cytochrome *c* location

HepG2 and Huh7 cells were plated on 18-mm cover glasses for 24 h and then treated with LAC117 (100 μg/mL). A mitochondrion-specific dye (MitoTracker Red FM: Molecular Probes Inc., Eugene, OR, cat.n.M22426) was added and incubated for another 30 min. The media were removed, and the cells were washed with PBS and fixed with an acetone: methanol solution for 5 min at − 20 °C. The fixed cells were washed with PBS for several times and incubated with cytochrome *c* antibody (Santa Cruz Biotechnologies, cat.n.13156) overnight at 4 °C. Subsequently, after washing with PBS several times, the cells were incubated with a mouse fluorescenct-labeled secondary antibody (1:100, Vector Laboratories, Burlingame, CA, cat.n.TI-2000) for 1 h at room temperature. The cells were stained with DAPI to visualize the nuclei. Finally, the cells were covered with a fluorescent mounting solution (Dako, Carpinteria, CA, cat.n.REF3023) before viewing with a confocal laser scanning microscope (Olympus, Tokyo, Japan).

### Tumor xenograft study

All animal experiments were performed in accordance with the guidelines of the INHA Institutional Animal Care and Use Committee (INHA IACUC) of the Medical School of Inha University (approval ID: INHA 150915–379-1). The cells (HepG2) were harvested and mixed in PBS. Six-week-old male BALB/c nu/nu mice (Orient Bio, Seoul, Korea) were inoculated with 1 × 10^7^ cells in the flank. When the tumor size reached approximately 50–100 mm^3^, mice were randomly divided into 2 groups (4 mice per group). Next, LAC117 (100 mg/kg) or vehicle (1% Tween 80) was administrated intraperitoneally once daily for 15 days. At the end of the 15 days treatment period, the animals were anaesthetized with mixture of ketamine (100 mg/kg) and xylazine (2%, 20 mg/kg). Tumor size was measured every 2 days, and it was calculated using the following formula: 0.5 × length × width^2^.

### Ex vivo organotypic spheroid culture

Male BALB/c nu/nu mice (4 weeks old, weighing 18–20 g) were obtained from Orient Bio. Animal Inc. (Seoul, Republic of Korea). The animals were fed standard rat chow and tap water ad libitum, and were maintained under a 12 h dark/light cycle at 21 °C. After one week of adaptation, Huh7 (8 × 10^6^ cells/mice) was inoculated into the right flanks of mice. When the tumor size reached approximately 300–500 mm^3^, they were surgically removed (*n* = 5). A 2-mm diameter section was excised and explanted on 2% agarose-coated 24 well plates with culture medium at 37 °C. After overnight incubation, the tumor spheroids were subjected to treatment with or without LAC117 (100 μg/mL) for 7 days. For IHC analysis, tissues were immediately fixed in 4% PFA overnight and paraffin-embedded slides were prepared for further analysis.

### Immunohistochemistry

Immunohistochemical staining of fixed paraffin-embedded specimens was performed using 8-μm-thick sections. Heat-induced epitope retrieval (HIER) was performed in a citrate buffer (pH 6.0) for 5 min before peroxidase quenching with 3% hydrogen peroxide (H_2_O_2_) in PBS for 10 min. The tissue sections were washed with PBS and blocked with normal goat or horse serum for 1 h and incubated at 4 °C overnight in 1:50 dilutions of primary antibodies against cleaved caspase-3, PCNA, p-AKT, and p-mTOR. The sections were then incubated with biotinylated secondary antibodies (1:100) for 1 h. The sections were visualized with an avidin-biotin peroxidase complex solution using an ABC kit (Vector Laboratories, cat.n.PK-6101), washed in PBS and developed with a diaminobenzidine tetrahydrochloride (DAB) substrate for 5 min, and then counterstained with hematoxylin. At least 3 randomly selected fields for each section were examined at x 200 magnification and analyzed.

### Statistical analysis

Data were presented as mean ± SEM, and were analyzed by an ANOVA and unpaired Student’s t-test. A *P*-value of 0.05 or less was indicated statistical significant. Comparisons of results were performed using a Student’s t-test.

## Results

### HPLC-MS analysis of LAC117

**Fig. 1:**
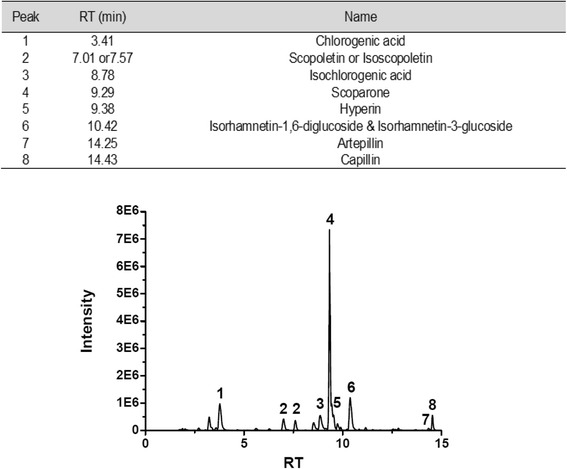
HPLC-MS chromatogram fingerprinting of LAC117

The protective effects of the AC against cancer have previously been reported (14). AC contains potent compounds as anti-inflammatory or anti-cancer components such as chlorogenic acid, esculetin, scopoletin, isochlorogenic acid, scoparone, hyperin, isorhamnetin-1,6-diglucoside, artepillin, and capillin, which are marker components of AC. Therefore, we identified whether LAC117 isolated from leaves of AC contains these components using HPLC-MS analysis. The chromatogram revealed the strong presence of the scoparone (RT = 9.29 min, 207 m/z; peak 4), chlorogenic acid (RT = 3.41 min, 355 m/z; peak 1), isorhamnetin-1,6-diglucoside (RT = 10.42 min, 625 m/z; peak 6), and hyperin (RT = 9.38 min, 465 m/z; peak 5, Fig. 1). The molecules responsible for these signals were identified by comparing them to the spectra and retention time of known standard compounds.

### LAC117 inhibits the proliferation of HCC cells

**Fig. 2:**
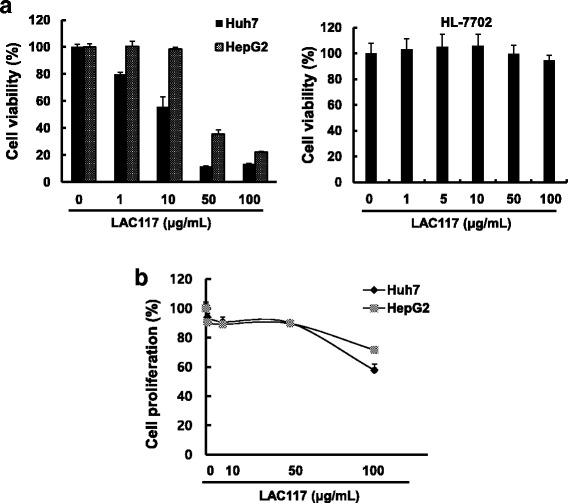
Effect of LAC117 on the growth and proliferation of HCC cells. **a** Huh7, HepG2, and HL-7702 cells were treated with LAC117 at the indicated concentration for 72 h, and then MTT assays were performed. **b** BrdU assay was performed to identify proliferation of HCC cells. Cells were treated with 10, 50, and 100 μg/mL of LAC117 for 24 h. Data are represented as means ± SEM of triplicates

To examine the effects of LAC117 on cell growth and viability, we performed the MTT assay using two HCC cell lines (HepG2 and Huh7). The cells were exposed to the indicated concentrations of LAC117 (1 to 100 μg/mL) for 72 h. LAC117 reduced cell viability of both HepG2 and Huh7 cells in a dose-dependent manner (Fig. 2a). In particular, LAC117 treatment inhibited cell growth by 60~ 90% at dose of 50 μg/mL depending on the cell type. To further evaluate this result, we determined cell proliferation by using the BrdU cell proliferation assay (Fig. 2b). In agreement with the MTT assay, LAC117 dose-dependently inhibited the proliferation of HCC cells. In the both studies, we found that Huh7 cells were more sensitive to LAC117 than HepG2 cells.

### LAC117 induces mitochondria-mediated apoptosis of HCC cells

**Fig. 3:**
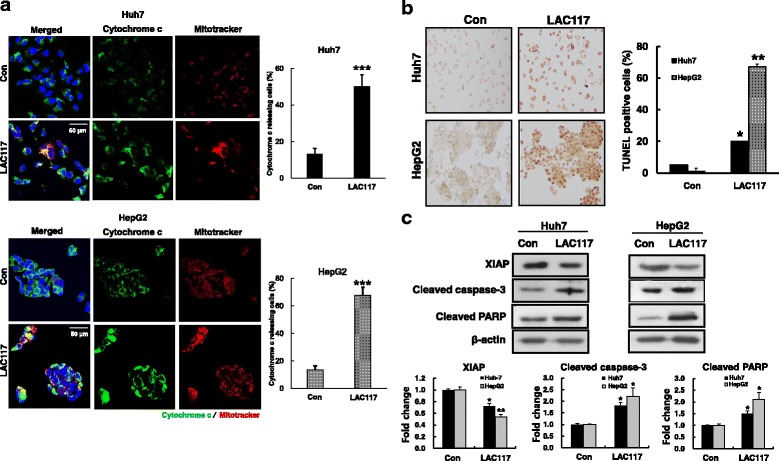
Effect of LAC117 on mitochondria-mediated apoptosis in HCC cells. **a** Huh7 and HepG2 cells were treated with LAC117 (100 μg/mL) for 6 h, and then stained with Mitotracker (green) and cytochrome *c* (red). Localization of cytochrome *c* in the cytosol was analyzed by confocal fluorescent microscopy at × 200 magnification. **b** Before performing TUNEL assay, HepG2 and Huh7 cells were treated with LAC117 (100 μg/mL) for 24 h. The results were observed by microscopy at × 200 magnification. **c** The expression of cleaved caspase-3, PARP, and XIAP was determined by Western blotting in cells treated with LAC117 (100 μg/mL) for 24 h. Data are represented as mean ± SEM of triplicates (*P < 0.05 and **P < 0.01*vs* Con)

During apoptosis, mitochondrial membrane potential regulates matrix configuration and the rapid release of cytochrome *c* from the mitochondrial intermembrane space into the cytosol. Therefore, we investigated whether LAC117 increases the release of cytochrome *c* through loss of mitochondria membrane potential. For this experiment, HepG2 and Huh7 cells were treated with LAC117 (100 μg/mL) for 6 h and then observed cytochrome *c* release into the cytosol by double staining with Mitotracker (green) and antibody against cytochrome *c* (red) in HCC cells. In this study, we observed that LAC117 significantly increased cytochrome *c* release which triggers the apoptotic process through caspase activation (Fig. 3a). We also investigated whether LAC117 could increase cleaved caspases 3/PARP, leading to apoptosis in HCC cells. When treated with LAC117 (100 μg/mL) for 24 h, the cells showed morphological features of apoptotic cells, such as DNA fragmentation by TUNEL staining. Also, the percentages of TUNEL-positive cells were increased in the LAC117-treated groups (Fig. 3b). We further found in western blotting study that treatment with LAC117 significantly increased the expression of apoptotic proteins (cleaved PARP and cleaved caspase-3), whereas the expression of anti-apoptotic protein (XIAP) was decreased in both cell lines, compared with control (Fig. 3c). These results indicate that LAC117 could induce mitochondria-mediated apoptosis in HCC cells.

### LAC117 inhibits PI3K/AKT pathway

**Fig. 4:**
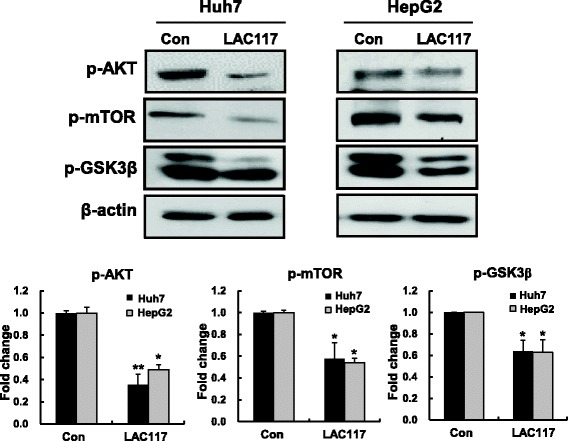
Effect of LAC117 on PI3K/AKT signaling pathway in HCC cells. To investigate the effect of LAC117 on PI3K/AKT signaling pathway, HepG2 and Huh7 cells were treated with 100 μg/mL of LAC117 for 1 h, and expression levels of p-AKT, p-mTOR, p-GSK3β, and β-actin were determined by Western blotting analysis. Data are represented as the mean ± SEM from triplicate experiments (*P < 0.05 and **P < 0.01*vs* Con)

Deregulated PI3K/AKT/mTOR signaling pathways are commonly found in HCC [20]. To understand the mechanism underlying the enhanced ant-cancer effect of LAC117, we investigated the inhibition of the PI3K/AKT signaling pathway in Huh7 and HepG2 cells after LAC117 (100 μg/mL) treatment for 1 h. LAC117 was observed to decrease the phosphorylation of AKT, mTOR, and GSK3β in Huh7 cells (Fig. 4).

### LAC117 increases apoptosis and inhibits cell proliferation in ex vivo tumor organotypic spheroids

**Fig. 5:**
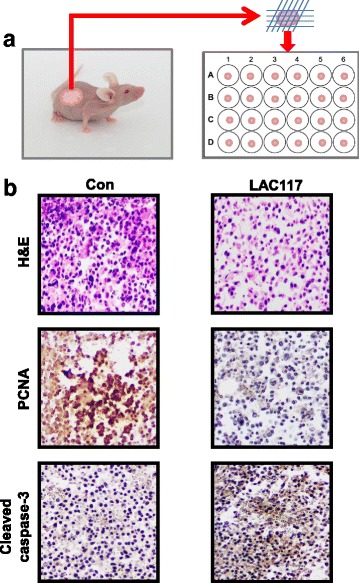
Effect on HCC tumor ex vivo models. **a** Huh7 xenograft tumors from Balb/c nude mice (*n* = 5) were harvested and cut into small pieces of ~ 2 mm, and each piece was maintained in culture media. Tumor spheroids cultured from the pieces were treated with LAC117 (100 μg/mL) for 7 days. **b** Tumor spheroids were excised and processed for immunostaining for PCNA, cleaved caspase-3, and H&E staining. The results were observed by microscopy at ;× 400 magnification

To further determine the apoptotic effect of LAC117, an ex vivo organotypic spheroid culture was established using xenograft-derived tumors in the Balb/c nude mice (Fig. 5a). As observed by H&E staining, apoptotic cells were significantly increased in the LAC117-treated group compared to that of the control group (Fig. 5b). Moreover, LAC117 treatment decreased the number of proliferating cells (PCNA-positive cells), but increased the number of apoptotic cells (cleaved caspase-3 positive cells, Fig. 5b). Taken together, our results show that LAC117 exhibit potent anti-cancer activity by inhibiting cell proliferation and inducing apoptosis.

### LAC117 inhibits tumor growth in vivo

**Fig. 6:**
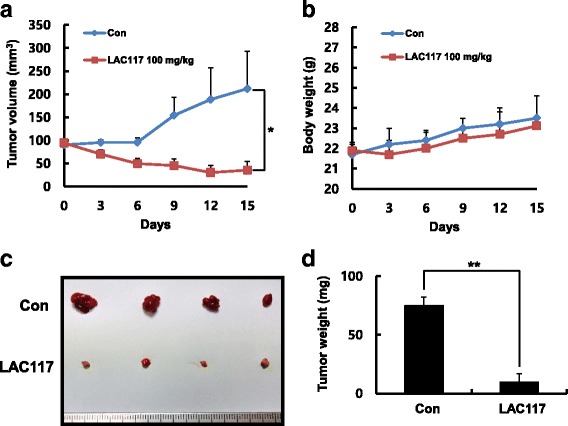
Tumor growth inhibition of LAC117 in HepG2 HCC xenograft models. **a**, **b** After the tumors reached 50–100 mm^3^ in size, the mice received an intraperitoneal administration of LAC117 (100 μg/mL) once daily for 15 days (*n* = 4, each group). Tumor volume and body weight were measured every 3 days in HepG2 HCC xenograft mouse models. The average tumor volume in the vehicle- or LAC117-treated group was plotted. **c**, **d** The tumors were isolated from HepG2 xenograft model, and were weighed immediately. Data are expressed as the mean ± SEM (*P < 0.05 and **P < 0.01)

**Fig. 7:**
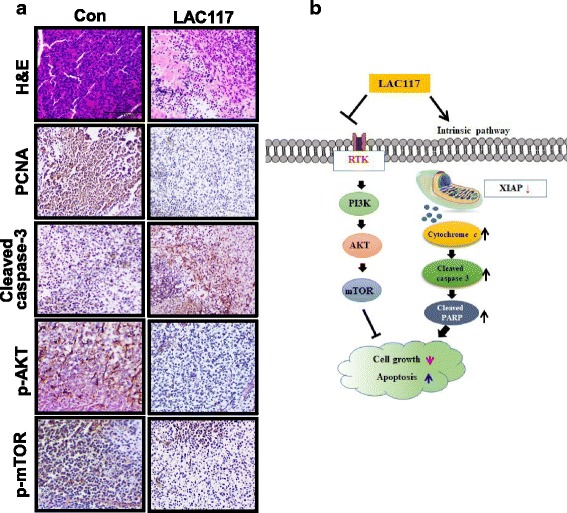
Effect of LAC117 on the proliferation and apoptosis in HepG2 xenografts. **a** Tumors were immunostained for PCNA, cleaved caspase-3, p-AKT, and p-mTOR including H&E staining. **b** Scheme for how LAC117 induces apoptosis and inhibits the growth of HCC cells. The results were observed by microscopy at × 400 magnification

To further assess whether LAC117 suppresses tumor growth in vivo, HCC xenograft models were used in Balb/c nude mice. After inoculation with HepG2 cells, mice were injected intraperitoneally with 100 mg/kg LAC117 for 15 days. As shown in Figs. 5c and 6a, LAC117 significantly reduced tumor volume and weight after 15 days. Moreover, no significant changes in body weight were observed in animals treated with LAC117 (Fig. 6b), showing that LAC117 was minimally toxic to mice at the curative dose. In histopathological analysis by H&E staining, we observed that there was a greater degree of tumor apoptosis and necrosis in the LAC117-treated group compared with the control group (Fig. 7). Also, LAC117 markedly decreased the expression of PCNA, a cell proliferation marker and increased that of cleaved caspase-3. In addition, LAC117 decreased the expression of p-AKT and p- mTOR, downstream of the PI3K/AKT pathways in tumor tissues. Experimental raw data were presented in Additional file 1.

## Discussion

Natural products have contributed to be most productive source in drug development. They provide the basis and inspiration for developing effective therapeutics for human diseases [21, 22]. In cancer, a lot of natural products have currently been used as cancer treatments and herbal medicines, food supplements, or nutraceuticals to alleviate toxicity of existing anticancer drugs. In particular, natural compounds have fundamental capacity for pharmacological treatments, and more than 50% of all anti-cancer drugs are derived from natural origins [23]. Recently, a lot of researches have shown that natural components are useful to prevent and to treat by targeting essential hallmarks in cancer [24]. Some natural herbal drugs are also developed to cure HCC [25].

*Artemisia capillaris* (AC) has been widely used as alternative therapy to treat various liver diseases including hepatitis in Asia [26]. In our study, we extracted an ethanol fraction (LAC117) from the dried leaves of AC because our preliminary study showed that the leaf extract had more potent anticancer activity compared to that of the entire AC plant, and investigated whether LAC117 has anti-cancer effect and its related mechanisms in HCC. Our study presented that LAC117 reduced cell growth and induced mitochondria-mediated apoptosis by inhibiting the PI3K/AKT pathway in vitro and in vivo.

Apoptosis is a physiological process responsible for cell suicide during development and tissue homeostasis. Thus, induction of apoptosis is good strategy in anticancer therapeutics. Also, it is involved in resistance to chemotherapy, contributing to the negative outcome of cancer treatments [27]. Upon apoptotic stimuli, mitochondria-mediated caspases activation triggers apoptosis in mammalian cells engage the cell death [28, 29]. In particular, cytochrome *c* is released from mitochondria into the cytosol, where it directly activates caspase caspase-3 activation [30, 31]. In this study, we observed that LAC117 enhanced the release of cytochrome *c* from the mitochondria to the cytoplasm, and increased the expression of cleaved caspase-3 and PARP, inducing apoptotic cell death. Furthermore, our in vivo results showed that LAC117 induced apoptosis in tumor xenograft models and significantly increased expression of cleaved caspase-3 together with decreased proliferation (PCNA) in tumor tissues. In previous studies, AC extracts induced apoptosis by the mitochondrial dysfunction in breast cancer and leukemia, which was similar to our findings [32, 33].

Given that LAC117 induced apoptosis in HCC, we attempted to find a main ingredient that would have the anticancer effect of LAC117. We detected scoparone, chlorogenic acid, hypersin, artepillin, and capillin, major components of AC using LC-MS, as previously reported [34]. Unfortunately, main components of LAC117 did not decrease cell proliferation and not induce high apoptosis compared with LAC117, extract of AC leaves in HCC cells (data not shown). Our results show that various ingredients of LAC117 could synergistically induce anti-cancer effect of LAC117 in HCC, which is characteristic of general natural products.

The PI3K/AKT pathway transduces signals from cell membrane receptors to the cytoplasm, and is closely related with the proliferation, growth, expansion, and metastasis of tumor cells [35, 36]. Recent researches have shown that PI3K/AKT pathway is improperly activated in various human cancers [37]. In particular, p-AKT expression has been reported to show a positive association with advanced tumor stage, invasion, and metastatic potential [38]. Some studies have reported that upregulation of the PI3K/AKT pathway has shown to be related to a poor prognosis in HCC and mTOR activation appears to be related with differentiated tumors and early recurrence after liver surgery [39, 40]. Therefore, the targeting of PI3K/AKT pathway may be effective to enhance chemotherapy in prevention of HCC. Various natural compounds such as resveratrol, curcumin have shown anti-cancer and anti-metastatic efficacy by suppression of PI3K/AKT signaling in HCC [41–43]. Recently, it has shown that the effect of natural compounds on HCC is not less than that of approved anti-cancer drugs [25, 44]. Indeed, Yun et al. have reported that acridine amine, extracted from sponges (species) effectively inhibited tumor growth compared with 5-FU by blockade of PI3K/AKT pathway in HCC [44]. Similar to previous studies, we found that LAC117 inhibited the activated PI3K/AKT signaling pathway via the decreases of p-AKT, p-mTOR, and p-GSK3β in HCC.

## Conclusions

Taken together, our results show that LAC117 exhibits potent anti-cancer activity by inhibiting cell proliferation and inducing apoptosis via regulation of the PI3K/AKT pathway in HCC. To the best of our knowledge, our results are the first to indicate that LAC117 might be a promising candidate as potentially useful anticancer drug against HCC.

## Additional file

Additional file 1: Raw data from cell viability, cytochrome *c* releasing cells, TUNEL positive cells, expression ratio of apoptosis/signaling molecules, and animal experiments. (XLS 64 kb)

## Abbreviations

AC: Artemisia capillaris Thunberg
EA: Ethyl acetate
HCC: Hepatocellular carcinoma
mTOR: mechanistic target of rapamycin
PCNA: Proliferating cell nuclear antigen
PI3K: Phosphoinositide 3-kinase
XIAP: X-linked inhibitor of apoptosis protein
